# Exosomes Derived from Human Urine-Derived Stem Cells Inhibit Intervertebral Disc Degeneration by Ameliorating Endoplasmic Reticulum Stress

**DOI:** 10.1155/2020/6697577

**Published:** 2020-12-07

**Authors:** HongFei Xiang, WeiLiang Su, XiaoLin Wu, WuJun Chen, WenBin Cong, Shuai Yang, Chang Liu, ChenSheng Qiu, Shang-You Yang, Yan Wang, GuoQing Zhang, Zhu Guo, DongMing Xing, BoHua Chen

**Affiliations:** ^1^Department of Orthopedics, The Affiliated Hospital of Qingdao University, Qingdao, China 266003; ^2^Cancer Institute, The Qingdao University, Qingdao, China 266003; ^3^Radiology Department, The Affiliated Hospital of Qingdao University, Qingdao, China 266003; ^4^Department of Orthopedics, Qingdao Municipal Hospital, Qingdao, Shandong 266011, China; ^5^University of Kansas, School of Medicine-Wichita, 929 N St. Francis Street, Wichita, KS, USA 67230; ^6^School of Life Sciences, Tsinghua University, Beijing, China 100084

## Abstract

**Objective:**

This study is aimed at determining the effects of human urine-derived stem cell-derived exosomes (USCs-exos) on pressure-induced nucleus pulposus cell (NPC) apoptosis and intervertebral disc degeneration (IDD) and on the ERK and AKT signaling pathways.

**Methods:**

The NPCs were obtained from patients with herniated lumbar discs. Western blot analysis (WB) and quantitative real-time polymerase chain reaction (qRT-PCR) were used to determine endoplasmic reticulum (ER) stress levels of NPCs under stress. Human USCs were identified using an inverted microscope, three-line differentiation experiments, and flow cytometry. A transmission microscope, nanoparticle size analysis, and WB procedures were used to identify the extracted exosomes and observe NPC uptake. A control group, a 48 h group, and a USCs-exos group were established. The control group was untreated, and the 48 h group was pressure-trained for 48 h, while the USCs-exos group was pressure-trained for 48 h and treated with USCs-exos. WB, qRT-PCR, and terminal deoxynucleotidyl transferase dUTP nick end labeling (TUNEL) analysis were used to determine the ER stress levels in stress conditions and after exosomal treatment. The AKT and ERK pathways were partially detected. Magnetic Resonance Imaging (MRI) and computed tomography (CT) were used to evaluate cell degeneration while exosomal effects on the intervertebral disc (IVD) tissue were determined by hematoxylin and eosin (HE) staining, Safranin O-fast green staining, immunohistochemical staining (IHC), nuclear magnetic resonance (NMR), spectrometric detection, and total correlation spectroscopy (TOCSY). IVD metabolites were also identified and quantified.

**Results:**

After pressure culture, ER stress markers (GRP78 and C/EBP homologous protein (CHOP)) in the NPCs were significantly elevated with time (*p* < 0.05). Human USCs are short and spindle-shaped. They can successfully undergo osteogenic, adipogenic, and chondrogenic differentiation. In this study, these stem cells were found to be positive for CD29, CD44, and CD73. The exosomes were centrally located with a diameter of 50-100 nm. CD63 and Tsg101 were highly expressed while the expression of Calnexin was suppressed. The exosomes can be ingested by NPCs. USCs-exos significantly improved ER stress responses and inhibited excessive activation of the unfolded protein response (UPR) as well as cell apoptosis and disc degeneration through the AKT and ERK signaling pathways (*p* < 0.05).

**Conclusion:**

Through the AKT and ERK signaling pathways, USCs-exos significantly inhibit ER stress-induced cell apoptosis and IDD under pressure conditions. It is, therefore, a viable therapeutic strategy.

## 1. Introduction

Lower back pain (LBP) is a common condition that affects the quality of life and results in a heavy financial burden [1]. The IDD is the main cause of LBP [2]. The IVD is a cartilage tissue composed of the central colloidal nucleus pulposus (NP), peripheral fibrous annulus (AF), and the upper and lower endplates [3]. During the IDD process, the NP area first exhibits degenerative changes. The apoptosis of NP cells is one of the most common manifestations of IDD [4–6]. Apoptosis refers to programmed cell death that is triggered by the activation of caspase [7]. Studies on IVD degeneration have focused on NPC apoptosis. Gruber and Hanley [8] were the first to identify apoptotic cells in the degenerated IVD. Later, Rannou et al. [9] proved that IDD is positively correlated with NP cell apoptosis. Therefore, the decrease in the number of cells in the nucleus pulposus area during IDD is the apoptosis of NPCs.

The biochemical cascade involved in IDD pathogenesis is extremely complex. IDD is associated with a decrease in proteoglycan content of NPCs, which leads to biomechanical changes [10]. However, the relationship between mechanical load and IDD onset has not been established. As it is subjected to mechanical stresses of different intensities in daily work and life, the IVD tissue plays an important role in spinal biomechanics [11]. Wilke et al. [12] measured the pressure on the L4/5 discs of healthy people. They found that the pressure is 0.1 MPa during sleep and 0.3-2.3 MPa during daily activities (it is 0.3-0.83 MPa when sitting, 0.5-1.1 MPa when standing, and 1.1-2.3 MPa when carrying a load). Zhang et al. [13] utilized 0.8 MPa to simulate IVD force during activity. And they found that it caused apoptosis in NPCs. Studies have documented that mechanical load is an important factor in IDD pathogenesis. Excessive mechanical pressure promotes cell apoptosis, increases the secretion of extracellular matrix-degrading enzymes, and suppresses the enzymes associated with the synthesis of the extracellular matrix, which ultimately lead to IDD [14, 15]. Therefore, studies should be aimed at elucidating the relationship between mechanical load and NPC apoptosis during IDD.

As an important organelle in eukaryotic cells, the ER is involved in the regulation of basic cellular processes, including folding transmembrane and secreted proteins, lipid synthesis, drug detoxification, Ca^2+^ storage, and signal transduction [16]. However, various factors such as inhibition of protein glycosylation, Ca^2+^ depletion, redox state changes, and the expression of misfolded proteins suppress ER functions. These dysfunctions may cause protein toxicity in the ER, collectively referred to as ER stress, which leads to the activation of the UPR [17, 18]. A certain degree of UPR protects cells from external stimuli and reestablishes cell homeostasis. However, under long-term or excessive pressure, UPR cannot restore protein homeostasis and cell homeostasis. Instead, cell death occurs through the ER stress-induced apoptosis mechanism [19–21]. Proper biomechanical loading plays an active role in the structure and function of articular cartilage [22]. However, continuous abnormal biomechanical stimulation leads to the accumulation of misfolded proteins in the ER lumen [23], causing ER stress [24].

Paracrine factors of mesenchymal stem cells (MSCs) play an important role in maintaining NP cell proliferation and inhibiting their apoptosis [25, 26]. MSCs secrete specific types of extracellular vesicles, such as exosomes, to achieve their therapeutic paracrine effect [27]. Exosomes are cell-secreted extracellular vesicles with a diameter of about 30-100 nm [28]. They provide a high number of biologically active substances, such as lipids, nucleic acids, and proteins, to recipient cells through membrane fusion. They are also involved in material and information exchange between cells [29]. However, MSC sources in the body are limited, and it causes certain trauma to the body when extracted, which limits its application. Human USCs exhibit a multidirectional differentiation potential. These cells have a wide range of sources from which they can be easily obtained in a safe and noninvasive manner. In addition, they are better sources of exosomes [30–32]. However, the specific mechanism by which exosomes promote the proliferation of NPCs and reduce their apoptosis has not been established.

In this study, we determined the expression levels of ER stress markers and UPR-related genes in NPCs under normal and stress conditions. The findings of this study provide new avenues for exploring the relationship between IDD and ER stress to inform the development of therapeutic options.

## 2. Materials and Methods

### 2.1. Isolation and Culture of NPCs

This study was approved by the Ethical Committee of the Affiliated Hospital of Qingdao University (approval number: QDFY-19-012-03). Patients or their guardians were required to sign informed consent before being enrolled in the study. The IVD tissues were obtained from patients with lumbar degenerative diseases who had been subjected to posterior foraminal lumbar intervertebral surgery. The AF and cartilaginous endplate (CEP) in the specimen were carefully removed under a microscope. After being washed 3 times with phosphate-buffered saline (PBS), the nucleus pulposus tissue was sliced to 1 mm^3^ and digested in 0.2% type II collagenase (Gibco) for 3 h. A 75 *μ*m filter was then used to remove tissue residues before the obtained cells were centrifuged at 800 r/min for 5-10 minutes. The cells were resuspended in Dulbecco's modified Eagle medium/F-12 (DMEM/F-12) medium (Gibco, Grand Island, NY, USA) supplemented with 10% fetal bovine serum (Gibco) and 1% penicillin-streptomycin. They were incubated at 37°C in a 5% CO_2_ environment.

### 2.2. USC Extraction

A 200 ml fresh sterile urine sample from 6 healthy male adults (average age 25.5 ± 1.26) was obtained in aseptic conditions. The urine sample was centrifuged at 400 g for 10 minutes, the supernatant was discarded, and the cell pellet was resuspended in PBS. It was centrifuged again at 200 g for 10 minutes, and the supernatant was carefully aspirated. The cell pellet was resuspended in 4 ml mixture of 10% fetal bovine serum (FBS) (Gibco, Australia), 1% penicillin-streptomycin, and REGM SingleQuot growth factor additive (Lonza, Basel, Switzerland) DMEM/F-12 medium (HyClone, Utah, USA). They were then inoculated in a 12-well plate that had been precoated with gelatin and incubated at 37°C in 5% CO_2_. The medium was changed every two days until a cell colony was formed. The colony was then transferred to RE/MC medium to continue the culture. The RE cell proliferation medium was made of 500 ml of RE cell basal medium with REGM SingleQuot kit components. The MC Proliferation Medium was a DMEM/F-12 medium supplemented with 10% FBS, 1% GlutaMAX (Gibco, Japan), 1% NEAA (Gibco, Grand Island, USA), 1% pen/strep (Gibco, Grand Island, USA), 5 ng/ml bFGF (PeproTech, Rocky Hill, USA), 5 ng/ml PDGF-AB (PeproTech, Rocky Hill, USA), and 5 ng/ml EGF (PeproTech, Rocky Hill, USA). The RE/MC medium was a 1 : 1 mixture of RE multiplication medium and MC multiplication medium. The passage was performed when the cell density reached 70%-80%. The P2-4 cells were obtained for subsequent experiments.

### 2.3. Flow Cytometry and Identification of USC Surface Markers

After digestion with trypsin, P3 generation USCs with good growth characteristics were obtained and washed 3 times using PBS after centrifugation. A cell suspension with a final concentration of 1 × 10^6^ cells/ml was made. A 100 *μ*l cell suspension was pipetted and mixed with 10 *μ*l of CD29, CD44, and CD73 (Santa Cruz Biotechnology, USA) monoclonal antibody working solution. They were incubated in the dark for 1 h at room temperature. The cells were washed 3 times and analyzed by flow cytometry.

### 2.4. Three-Line Differentiation of USCs

To evaluate the differentiation potential of human USCs, they were induced to differentiate into osteogenic, adipogenic, and chondrogenic cells according to the osteogenesis, adipogenesis, and chondrogenesis differentiation culture kit (Cyagen Biosciences, Guangzhou, China) instructions. USCs were seeded into a 6-well plate. When the cell fusion rate was 80%, differentiation was induced. Upon the induction of osteogenic differentiation, osteogenic differentiation medium (Cyagen, Guangzhou, China) was added and replaced every 3 days. After 21 days of induction, the cells were fixed in 4% paraformaldehyde and stained with Alizarin Red for observation. The osteogenic differentiation complete medium kit contains 175 ml basal medium, 20 ml serum, 2 ml penicillin-streptomycin, 2 ml glutamine, 2 ml beta-glycerophosphate sodium, 400 *μ*l ascorbic acid, and 20 *μ*l dexamethasone. To induce differentiation by adipogenesis, the adipogenic differentiation medium A (Cyagen, Guangzhou, China) was added; 3 days later, it was changed to adipogenic differentiation medium B (Cyagen, Guangzhou, China); 24 h later, it was again changed to medium A. Medium alterations were made for a total of 3 times. The cells were finally fixed with 4% paraformaldehyde and observed after staining with Oil Red O. The adipogenic differentiation medium A kit contains 175 ml basal medium, 20 ml fetal bovine serum, 2 ml penicillin-streptomycin, 2 ml glutamine, 400 *μ*l insulin, 200 *μ*l 3-isobutyl-1-methylxanthine (IBMX), 200 *μ*l dexamethasone, and 200 *μ*l rosiglitazone. The adipogenic differentiation medium B kit contains 175 ml basal medium, 20 ml fetal bovine serum, 2 ml penicillin-streptomycin, 2 ml glutamine, and 400 *μ*l insulin. Upon the induction of chondrogenesis, cell counting was done. Approximately 2.5 × 10^5^ of USCs were centrifuged at 150 g in a 15 ml centrifuge tube for 5 minutes, and the supernatant was discarded. Chondrogenic differentiation medium (0.5 ml) was added to the cells and cultured. The medium was changed every 3 days. The cells were then fixed in 4% paraformaldehyde for 21 days and sliced after embedding in paraffin. They were finally stained with Alcian Blue for observation. The chondrogenic differentiation medium kit contains 194 ml of basic medium, 600 *μ*l ascorbic acid, 20 *μ*l dexamethasone, 2 ml ITS supplement (ITS+supplement), 200 *μ*l sodium pyruvate, 200 *μ*l proline, and 2 ml transforming growth factor-*β*3 (TGF-*β*3).

### 2.5. Extraction and Identification of Exosomes

After the cells had grown to a 70-75% confluence, the medium was aspirated. The cells were washed 3 times using PBS. Serum-free medium was added to the cells and cultured for 48 h. The culture medium was then centrifuged at 500 g for 10 min at 4°C to remove residual cells, centrifuged at 2000 g for 20 min at 4°C to remove cell debris, and centrifuged at 10000 g for 30 min at 4°C to further remove impurities. The supernatant was obtained and filtered using a 0.22 *μ*m filter membrane to remove oversized particles. The supernatant was then ultracentrifuged at 100000 g at 4°C for 2 h. The resulting pellet was resuspended in PBS. A transmission electron microscope (TEM) (JEM-1200EX, Japan) was then used to observe the morphology of exosomes while the NanoSight detector (Malvern, England) as well as the NTA detection and analysis software was used to analyze the number and size distribution of the exosomes. Western blot was used to detect exosomal markers (CD63, TSG101, and Calnexin).

### 2.6. Uptake of Exosomes by NPCs

The PKH26 fluorescent dye kit (Sigma-Aldrich) was used to label exosomes according to the manufacturer's instructions. Excess dye was neutralized with an equal volume of PBS containing 5% BSA. The labeled cells were then centrifuged at 4°C and 100000 g for 70 minutes. The supernatant was removed, and the labeled cells were resuspended in 50 *μ*l PBS. The prepared exosomes of labeled USCs were added to NPCs, incubated for 12 h in the dark, fixed in 4% paraformaldehyde for 20 minutes, stained with DAPI, and mounted with glycerol to observe their uptake using a laser confocal microscope. The Leica Application Suite Advanced Fluorescence software was used to analyze the obtained image.

### 2.7. Construction of Pneumatic Pressure Model

The NPCs were introduced into a pneumatic pressure device assembled by our research group to simulate pressure-associated IVD tissue damage. The device is composed of a cell culture apparatus and a high-pressure gas scheme. About two liters of double-distilled water was added to the cell culture device to a level that did not exceed the cell placement equipment. After placing the cell culture plate in the corresponding position, the equipment was turned off. To obtain 1.0 MPa, a high-pressure gas device was used to fill the cell culture environment with a mixed gas containing 90% N_2_, 5% CO_2_, and 5% O_2_. The temperature of the cell culture device was set to 37°C. After 48 hours of culture, the cells were obtained for Western blotting and other experiments.

### 2.8. Western Blot Analysis

The obtained cells were lysed in radioimmunoprecipitation assay (RIPA) lysis buffer (Solarbio, Beijing, China) containing 1 mM phenylmethanesulfonyl fluoride (PMSF) and protease inhibitors to extract proteins. The concentration of the extracted protein was determined using a bicinchoninic acid (BCA) kit (Solarbio, Beijing, China). The protein and the loading buffer were then mixed at a ratio of 4 : 1 (*V*/*V*), boiled for 10 minutes, separated by sodium dodecyl sulfate-polyacrylamide gel electrophoresis (SDS-PAGE), and transferred to a polyvinylidene fluoride (PVDF) membrane. The PVDF membrane was sealed with 5% skimmed milk powder at room temperature. The membrane was then incubated overnight at 4°C with primary antibodies (CD63, TSG101, Calnexin, GRP78, CHOP, GRP94, caspase-3, caspase-12, p-PERK, PERK, ATF6, p-IRE1*α*, IRE1*α*, XBP1, ATF4, ERK, p-ERK, AKT, p-AKT, and *β*-actin) (Santa Cruz Biotechnology, USA). After overnight incubation, the membrane was incubated with a horseradish peroxidase- (HRP-) labeled secondary antibody (ABclonal, Wuhan, China) for 1 h. An ECL kit (Thermo Fisher Scientific, Rockville, MD, USA) was then used for luminescence observation. The obtained images were analyzed using Image Lab software (Bio-Rad, Hercules, CA, USA).

### 2.9. Quantitative Real-Time Polymerase Chain Reaction (qRT-PCR)

Total RNA was extracted from the cultured cells using a TRIzol reagent (Invitrogen, Carlsbad, CA, USA). Reverse transcription and gene amplification procedures were done according to the kit manufacturer's instructions (TransGen Biotech, Beijing, China). GAPDH was set as an internal reference. The primer sequences used in this study are presented in Table 1. The obtained data was analyzed using the 2-*ΔΔ*Ct algorithm.

### 2.10. Terminal Deoxynucleotidyl Transferase dUTP Nick End Labeling (TUNEL) Staining

TUNEL staining was used to detect cell apoptosis. Cells were fixed in 4% paraformaldehyde for 1 h at room temperature. They were then incubated with 0.5% TritonX-100 in PBS for 5 minutes. After washing using PBS, they were incubated for 60 minutes as described by the apoptosis detection kit (Roche, Basel, Switzerland). The cells were finally stained with 0.1 g/ml DAPI and mounted with glycerol to observe apoptosis using a laser confocal microscope.

### 2.11. Rat Tail Degeneration Experiment

Twenty 3-month-old SD rats were purchased for the in vivo experiments. Five rats were randomly selected and assigned to the normal control group, without any treatment; the remaining 15 rats were all treated as the experimental group. The rats were anesthetized with 2% pentobarbital, and the three IVDs (Co4/5, Co5/6, and Co6/7) of each rat were determined on the tail vertebrae by palpation. A 21 G needle was used to puncture the IVDs of Co4/5 and Co5/6, respectively. The Co4/5 was injected with USCs-exos (100 *μ*g/ml). The injection was done every 2 weeks. The condition of the IVD was observed in the 4^th^ and 8^th^ weeks using CT and MRI scans. Then, the degeneration of the IVD was evaluated according to the signal changes in MRI images. After the 8^th^ week, the rats were sacrificed and the IVD samples were obtained. This experimental protocol was approved by the Animal Experiment Committee of Qingdao University, China.

### 2.12. Safranin O-Fast Green Cartilage Stain

The IVD tissue samples were fixed with paraformaldehyde, decalcified, dehydrated, and then embedded in paraffin. The Safranin O-Fast Green Cartilage Staining Kit (Solarbio, Beijing, China) was used for staining according to the manufacturer's instructions. The tissue samples were deparaffinized in water, introduced in freshly prepared Weigert dye solution for 3-5 minutes, and washed with water. They were differentiated in acidic differentiation solution for 15 seconds, washed with distilled water for 10 minutes, immersed in the fast green staining solution for 5 minutes, and quickly washed using a weak acid solution for 10-15 seconds to remove the excess fast green. The samples were then introduced into Safranin O stain for 5 min, dehydrated using 95% ethanol and absolute ethanol, and made transparent using xylene. They were observed after sealing using an optical resin.

### 2.13. Hematoxylin-Eosin (HE) Staining

The samples were decalcified and fixed in formaldehyde, dehydrated, embedded in paraffin, and sectioned. HE staining was performed using the Hematoxylin and Eosin (HE) Staining Kit (Solarbio, Beijing, China) according to the manufacturer's instructions. Briefly, paraffin sections were deparaffinized, hydrated, stained using the hematoxylin staining solution for 5-20 minutes, and introduced into the differentiation solution for 30 seconds. The sections were washed with warm water at 37°C, introduced in eosin dye solution, washed, soaked again, dehydrated, made transparent using xylene, mounted, sealed with neutral gum, and observed under a microscope.

### 2.14. Immunohistochemical Analysis (IHC)

The rats were euthanized, and their IVDs were obtained, decalcified, fixed in formaldehyde, dehydrated, and embedded in paraffin. The sections were dewaxed, hydrated, incubated at room temperature with 3% hydrogen peroxide for 10 minutes, and then twice soaked in PBS for 5 minutes each time for antigen repair. The sections were then blocked using PBS supplemented with 5% goat serum for 1 h. Caspase-3 primary antibody (Santa Cruz Biotechnology, USA) was then added and incubated overnight at 4°C. After overnight incubation, the sections were rinsed with PBS 3 times; 5 minutes later, a horseradish peroxidase-labeled secondary antibody was added and incubated at 37°C for 30 minutes. The sections were thrice rinsed using PBS for 5 minutes each time. Approximately 5 ml of diaminobenzidine (DAB) was added for 3-15 minutes to induce color development. Tap water was then used to fully rinse after which the sections were counterstained, dehydrated, made transparent using xylene, covered with neutral resin, and observed under a microscope.

### 2.15. Nuclear Magnetic Resonance Spectroscopy and TOCSY Spectra Identification of Metabolites in IVD

Fresh IVD tissue was ground and lysed in RIPA lysis buffer (Solarbio, Beijing, China) containing 1 mM phenylmethanesulfonyl fluoride (PMSF) and protease inhibitors to obtain protein. The concentration of the extracted protein was detected using a BCA kit (Solarbio, Beijing, China) according to the manufacturer's instructions. Five hundred microliter of D_2_O and 100 *μ*l of 10% 3-(trimethylsilyl) propionic acid sodium salt (TSP) were then added to the protein. They were mixed and centrifuged at 14,000 g for 15 minutes (to remove the precipitated particles of the tissue in the solution). 550 *μ*l of the supernatant was obtained and used to detect the protein and metabolite solution using the 600 MHz NMR (Bruker, Germany) equipment according to the manufacturer's instructions. The characteristic TOCSY, two-dimensional spectrum of the tissue metabolites and protein solution, was obtained and analyzed using the MestReNova (Mestrelab Research Co. Ltd., USA) software in order to identify the main differences in the metabolite and protein residues.

### 2.16. Statistical Analysis

The experiments were done in triplicates. Continuous data is expressed as the mean ± standard deviation (SD) while the nonparametric data is expressed in the median and interquartile range. One-way analysis of variance (ANOVA) was used to compare the statistical differences among groups while the parallel group parameters were compared by a *t*-test. *p* < 0.05 was set as the threshold for statistical significance. Statistical analysis was done using SPSS 20.0 software (SPSS, Chicago, IL, USA), while the GraphPad Prism 8 (GraphPad Software, USA) software was used to draw statistical graphs.

## 3. Results

### 3.1. Expression of ER Stress Markers in NPCs after Pressure Culture

**Figure 1:**
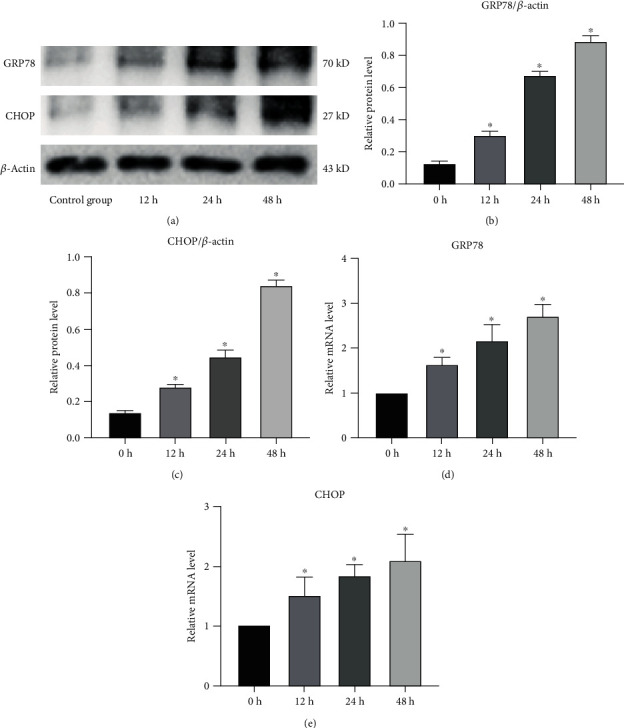
Endoplasmic reticulum stress level after stress culture of human NPCs. (a–c) Analysis of the protein levels of GRP78 and CHOP by Western blot (a), and based on this, the gray values of relative protein expression are compared (b, c). *β*-Actin was used as an internal control. ^∗^Compared to the control group, *p* < 0.05. (d, e) The mRNA levels of GRP78 and CHOP in NPCs after pressure culture. ^∗^Compared to the NC group, *p* < 0.05.

Endoplasmic reticulum stress is associated with the pathogenesis of IDD. Severe ER stress induces excessive apoptosis of NP cells [33, 34]. In this study, protein was extracted from the pressure-cultured NPCs. The ER stress marker GRP78 and the downstream protein CHOP were detected by Western blot (Figures 1(a)–1(c)). The expression levels of GRP78 and CHOP were also determined by qRT-PCR at different times of pressure culture (*n* = 3). The gene and protein expressions of GRP78 and CHOP were positively correlated with the time of pressurized culture (Figures 1(d) and 1(e)). These findings indicate that external pressure stimulation induced ER stress, which caused NPC apoptosis thereby leading to the occurrence or acceleration of IDD.

### 3.2. Identification of USCs-Exos and NP Cell Uptake

**Figure 2:**
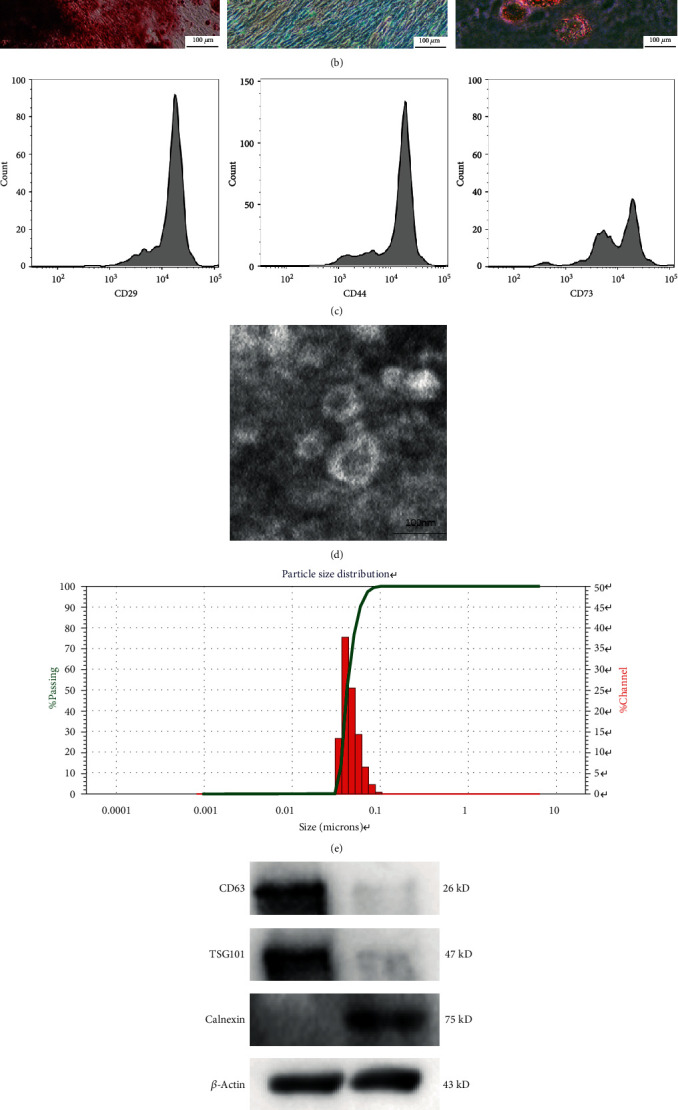
Identification of human USC exosomes (USCs-exos). (a) Human USCs exhibit a short fusiform or spindle-shaped appearance, with sporadic cells appearing on day three (left picture) and large numbers of cells appearing at day seven (right picture). (b) Osteogenic, adipogenic, and chondrogenic differentiation capabilities of USCs were determined by Alizarin Red, Oil Red O, and Alcian Blue staining. (c) USC surface markers (CD29, CD44, and CD73) were detected by flow cytometry. (d) A typical image of USCs-exos morphology as obtained by a transmission electron microscope (TEM). (e) Particle size distribution of USCs-exos as determined by the nanoparticle size analysis. (f) The protein marker of USCs-exos as detected in exosomes and USCs by Western blot analysis. (g) Exosomes taken by NP cells incubated with PKH26-labeled USCs-exos for 12 hours, and NP cell nuclei were stained with DAPI.

Human USCs were extracted from the urine of healthy adults. Under a light microscope, human USCs exhibit a short fusiform or spindle-shaped appearance (Figure 2(a)). Their differentiation into three cell lines was induced by osteogenic, cartilage, and adipogenic media (Figure 2(b)). Flow cytometry showed that the USC surface markers CD29, CD44, and CD73 were positive (Figure 2(c)). USCs exhibit a variety of biological characteristics such as clonogenicity, expression of specific cell surface markers, and pluripotent differentiation abilities that correspond to those of the adult MSCs [28, 35–37]. The positive CD29, CD44, and CD73 findings were consistent with previous studies. The shape of USCs-exos was similar to a circular sacculus with a depressed center with a diameter in the range of 50-100 nm (Figure 2(d)). Nanoparticle size analysis revealed that the USCs-exos exhibited a particle size range of 50-100 nm (Figure 2(e)). The USC exosomal marker proteins (CD63 and Tsg101) were highly expressed, while the negative protein Calnexin was suppressed (Figure 2(f)). The exosomes were labeled with PKH26 and incubated with NP cells to confirm that they could be taken up by NPCs. Fluorescently labeled exosomes were observed in the cytoplasm of NPCs, indicating that the exosomes had been taken up by NPCs (Figure 2(g)).

### 3.3. USCs-Exos Inhibit Pressure-Induced ER Stress and Suppress NPC Apoptosis

**Figure 3:**
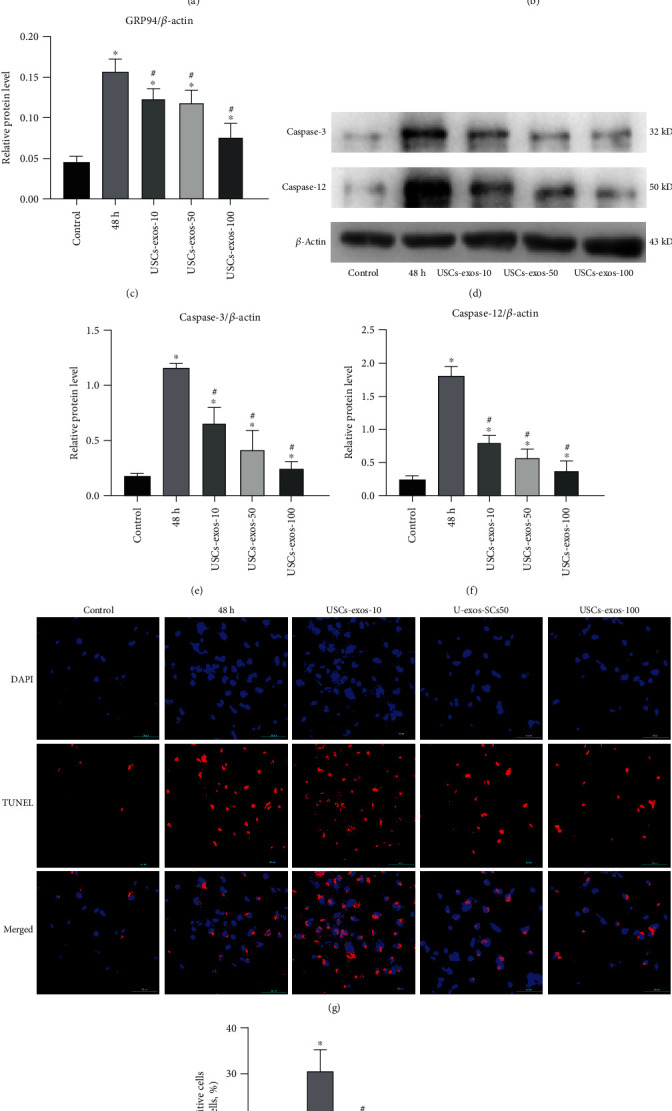
Under stress conditions, USCs-exos suppressed the expression of GRP78 and GRP94. Except the control group, NP cells were cultured for 48 h under pressure. USCs-exos-10, 50, and 100 indicated that 10, 50, or 100 *μ*g/ml exosomes were added to each response group. (a) Protein levels of GRP78 and GRP94 were measured by Western blot analysis, and their relative quantities were calculated (b, c) using *β*-actin as an internal reference. (d) Western blot analysis was used to detect the expression levels of caspase-3 and caspase-12, and their relative quantities (e, f) were calculated using *β*-actin as an internal reference. (g) Fluorescence images of TUNEL analysis in different groups. The nucleus was stained with DAPI. (h) The proportion of apoptotic cells according to TUNEL staining. Data are expressed as the mean ± SD. ^∗^Compared to the control group, *p* < 0.05; ^#^compared to the 48 h group, *p* < 0.05.

Pressure stimulation causes cell apoptosis [38, 39]. Stem cell exosomes exhibit an antiapoptotic effect under a variety of conditions [25, 27]. Figures 3(a)–3(c) show the expression levels of ER stress marker proteins (GRP78 and GRP94) and their relative protein expression levels. Severe ER stress causes cell apoptosis; therefore, the expression of caspase-3 and caspase-12 between the groups was compared (Figures 3(d)–3(f)). It was revealed that the apoptotic rate increased under pressure culture conditions while treatment with USCs-exos decreased the apoptotic rate (Figures 3(g) and 3(h)). The above results indicate that pressure elevates the ER stress of NPCs, while USCs-exos inhibit the occurrence of ER stress and, therefore, reduce cell apoptosis, which may exert a protective effect on the IVD tissue.

### 3.4. USCs-Exos Inhibit the Activation of UPR Caused by ER Stress in Human NPCs under Pressure Culture Conditions

**Figure 4:**
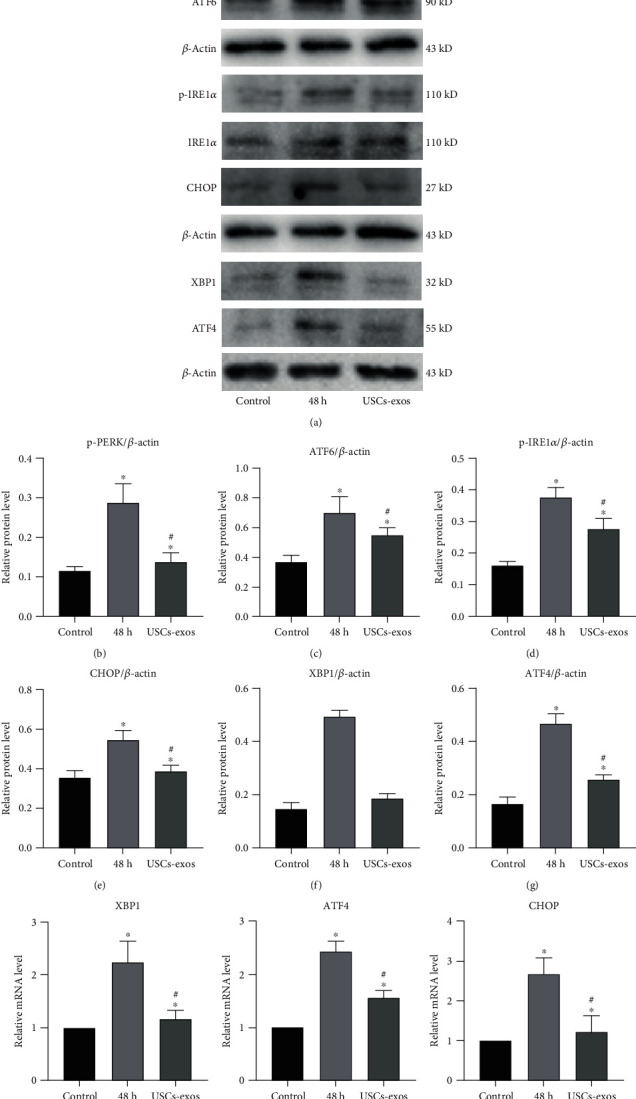
USCs-exos enhance the activation of UPR and related proteins under stress conditions. The USCs-exos group was treated with USCs-exos (100 *μ*g/ml). (a–g) Protein expression levels of p-PERK, PERK, ATF6, p-IRE1*α*, IRE1*α*, XBP1, ATF4, and CHOP as determined by Western blot analysis and calculation of their relative quantities (b–g) using *β*-actin as an internal reference. (h–j) The transcription levels of XBP1 (h), ATF4 (i), and CHOP (j) as determined by qRT-PCR. The data are expressed as the mean ± SD. ^∗^Compared to the control group, *p* < 0.05; ^#^compared to the 48 h group, *p* < 0.05.

It has been documented that severe or prolonged ER stress may overactivate UPR, excessive protein degradation, and eventually apoptosis [40]. The role of USCs-exos in stress-induced ER stress was determined by evaluating the expression levels of three transmembrane proteins (protein kinase-like endoplasmic reticulum kinase (PERK), inositol-requiring protein 1*α* (IRE1*α*), and activating transcription factor (ATF6)) in the classic branch of UPR (Figure 4(a)). Pressure culture enhanced the expression levels of ATF6, phosphorylated IRE1*α* (p-IRE1*α*), and phosphorylated PERK (p-PERK) in NPCs, indicating that UPR was activated (Figure 4(a)). Furthermore, transcription of the downstream genes of UPR gene activating transcription factor 4 (ATF4) and X-box binding protein 1 (XBP1) was also elevated (Figures 4(h) and 4(i)). After stress culture, CHOP was found to be activated at the RNA and protein levels as an apoptotic mediator of ER stress (Figure 4(j)). Compared to the control group, the expression levels of ATF4, XBP1, and CHOP in the exosomal group were significantly suppressed. The above results indicate that USCs-exos regulate URP activation by regulating the ER stress of NPCs under pressure.

### 3.5. USCs-Exos Inhibit Stress-Induced ER Stress-Related Apoptosis through the AKT and ERK Pathways

**Figure 5:**
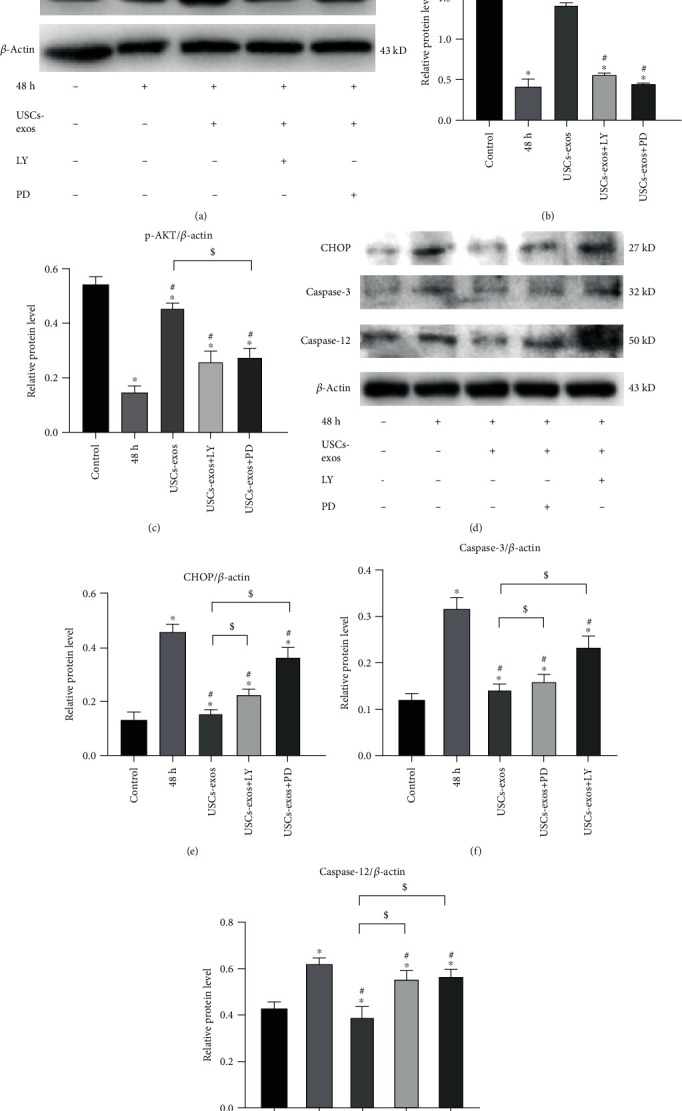
USCs-exos regulate ER stress under stress-induced conditions by activating the AKT and ERK signaling pathways in NPCs. In the USCs-exos group, USCs-exos (100 *μ*g/ml) was added for intervention under pressure. (a–c) Protein levels of AKT, p-AKT, ERK, and p-ERK were evaluated by Western blotting, and their relative quantities were calculated (b, c) using *β*-actin as the internal reference. LY294002 (LY) is an inhibitor of PI3K/AKT. PD98059 (PD) is an inhibitor of ERK1/2 phosphorylation. (d–g) The protein levels of CHOP, caspase-12, and caspase-3 were measured by Western blotting and statistically analyzed (e–g) using *β*-actin as an internal control. The data are expressed as the mean ± SD. ^∗^Compared to the control group, *p* < 0.05; ^#^compared to the 48 h group, *p* < 0.05; ^$^compared to USCs-exos group, *p* < 0.05.

The uptake of exosomes by cells activates the AKT and ERK pathways [41, 42]. In this study, we found that after pressure incubation, the phosphorylation levels of AKT and ERK in the USCs-exos (100 *μ*g/ml) group were significantly elevated when compared to the control group (Figures 5(a)–5(c)). Figures 5(d)–5(g) show the expression levels of CHOP and the activation of caspase-3 as well as caspase-12. It was observed that under pressure, the phosphorylation levels of AKT and ERK decreased. This was attributed to the expression of CHOP and the activation of caspase protein. Treatment of the pressure-cultured cells with USCs-exos significantly activated the AKT and ERK signaling pathways, thereby suppressing CHOP expression while downregulating caspase-3 and caspase-12 activation. When the NPCs were treated with AKT signal inhibitor (LY294002) or ERK signal inhibitor (PD98059), the protective effect of USCs-exos was reduced. These experiments imply that USCs-exos partially activate AKT and ERK signal transduction pathways in human NPCs to inhibit the related ER stress-induced apoptosis.

### 3.6. USCs-Exos Inhibit ER Stress-Related Apoptosis and Suppress IVD Degeneration

**Figure 6:**
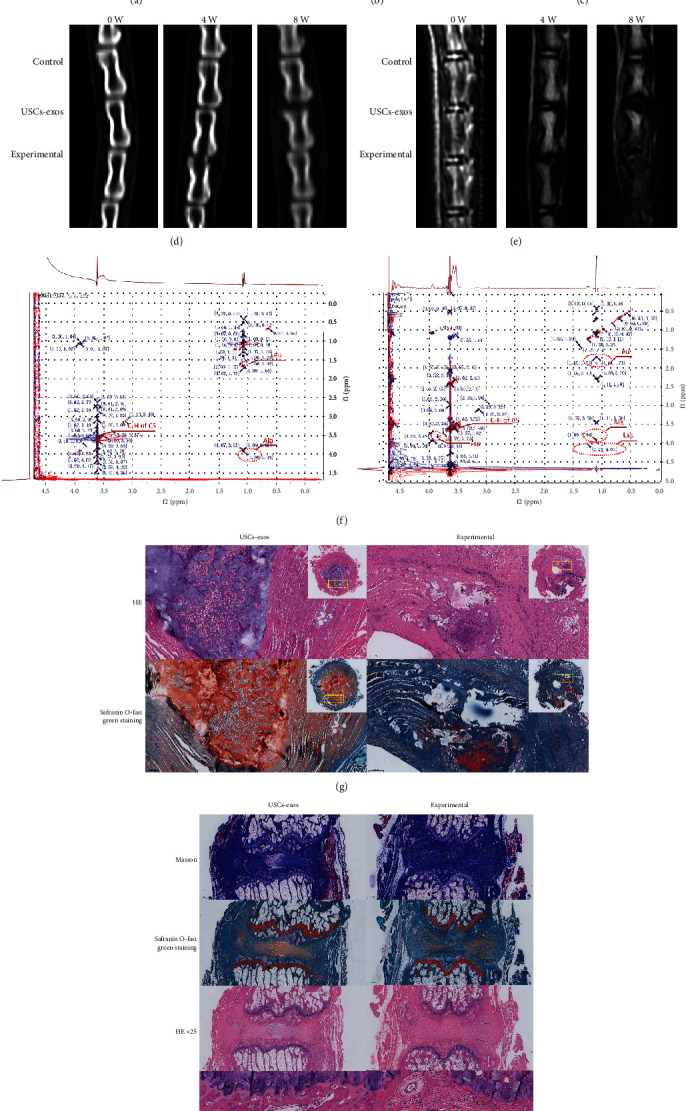
USCs-exos inhibits ER stress-associated cell apoptosis and delays IDD in vivo. (a–c) The expression levels of GRP78 and CHOP in rat degeneration models as determined by Western blotting, and their relative quantities (b, c). (d) CT scans of the three adjacent IVDs of the rat's tail vertebra at 0, 4, and 8 weeks. The height of the intervertebral space in the experimental group was significantly lower than that of the USCs-exos group and the control group. (e) T2W1-weighted images of the MRI scans performed on the rat's tail at 0, 4, and 8 weeks. The degeneration of the experimental group was significantly stronger than that of the USCs-exos group and control group. (f) In the NMR detection, compared to the simple puncture, the CHOP amino acid residue leucine (Leu) in the IVDs punctured and injected with USCs-exos was significantly suppressed; caspase-3 amino acid residue aspartic acid. The content of the injected exosomes in the IVD is significantly lower than that of the pure puncture segment; lactic acid (Lac) levels were also significantly low. (g) The HE and Safranin O-fast green staining of the IVD revealed that the IVD with simple puncture was more disordered and looser than the annulus fibrosus injected with USCs-exos and contained a large number of inflammatory cells and scars. The degenerated nucleus pulposus tissue protrudes into the annulus fibrosus. (h) Masson staining, Safranin O-fast green staining, and HE staining all showed that the degree of degeneration of the IVD tissue of injected USCs-exos was less, and IHC showed that the expression of caspase-3 was lower. ^∗^Compared to the control group, *p* < 0.05; ^#^compared to the USCs-exos group, *p* < 0.05.

Figures 6(d) and 6(e) show the MRI and CT scans of the rat models at 0, 4, and 8 weeks after the puncture to measure the disc height and degeneration grade. The USCs-exos were shown to delay disc degeneration by reducing apoptosis (Figures 6(a)–6(c)). In addition, the characteristic TOCSY two-dimensional spectra of the metabolites and protein solutions of IVD tissues also showed obvious changes (Figure 6(f)). Compared to the simple puncture, the CHOP amino acid residue leucine (Leu) in the IVDs punctured and treated with USCs-exos was significantly reduced; the quantity of aspartic acid (Asp), the amino acid residue of caspase-3, in the IVD of the injected exosomes was significantly lower than that of the pure puncture segment; after injection of the exosomes, the anaerobic glycolysis product, lactic acid (Lac), was significantly decreased. The HE and Safranin O-fast green staining of cross-section of IVD tissue showed (Figure 6(g)) that the IVD with a simple puncture was more disordered and looser than the annulus fibrosus injected with USCs-exos. Moreover, it contained a lot of inflammatory cells and scars with degenerating nucleus pulposus tissue protruding into the annulus. In short, the degeneration degree was significantly higher. And on the longitudinal section of the IVD tissue (Figure 6(h)), Masson staining showed that the fibrous tissue content of the IVD in the simple puncture group was significantly higher than that of the USCs-exos group, while the nucleus pulposus tissue content was significantly lower than that of the USCs-exos group. Similar results were observed with HE staining and Safranin O-fast green staining. These findings were confirmed by the immunohistochemical staining procedure. Compared to the simple puncture group, caspase-3 expression, which is associated with apoptosis, was significantly suppressed after treatment with USCs-exos (Figure 6(h)).

Therefore, the in vivo study revealed that USCs-exos inhibit IDD by inhibiting ER stress-associated cell apoptosis.

## 4. Discussion

Studies have established that paracrine plays an important role in stem cell-associated inhibition of IDD [43, 44]. Exosomes are the key bioactive paracrine components of stem cells and can replace stem cell transplant-based therapies. Excess stress due to mechanical loads and ER stress is involved in IDD pathogenesis. Abnormal mechanical load-associated pressures can lead to the apoptosis of NPCs [13, 39]. Long-term abnormal pressure enhances ER stress, which leads to cell apoptosis. The earliest response to ER stress is UPR. Long-term excessive stress leads to a transition from adaptive to proapoptotic responses, which cause pathological conditions [45]. The activation of UPR triggers a series of downstream cascade reactions, including ATF4 and XBP1, which enhance the overexpression of CHOP [46–49]. Overexpressed CHOP activates caspase-3 and elevates the apoptotic rate [50].

In this study, USCs-exos were shown to inhibit NPCs ER stress-induced cell apoptosis in a dose-dependent manner. The antiapoptotic effect of USCs-exos is realized through the inhibition of ER stress and the activation of the AKT and ERK signaling pathways. After prolonged stress stimulation, NPCs exhibit excess ER stress that leads to UPR accumulation. UPR accumulation elevates the secretion of CHOP protein that cleaves caspase-12 and caspase-3. In addition, a modified gas pressurization device was used to cultivate NPCs. As the pressurization time increased, the expression levels of GRP78 and CHOP increased. Furthermore, the expression levels of caspase-12 and caspase-3 were also elevated. Treatment of NPCs using USCs-exos revealed that as the exos concentration increased, the expression levels of CHOP, caspase-3, and caspase-12 decreased. This indicates that USCs-exos inhibited NPC apoptosis by inhibiting ER stress in a dose-dependent manner. Therefore, we hypothesized that stress stimulation promotes IDD progression through ER stress. Inhibiting ER stress may, therefore, be an effective way of delaying or reversing IDD.

Stem cells are considered to be ideal for IVD regeneration because they can prevent IVD cells from aging and apoptosis [51]. Studies have found that exosomes can be used among different individuals or species and are, therefore, viable alternatives to stem cell transplantation therapy [29, 52, 53]. The effects of MSCs-exos on immune regulation, wound healing, inflammation, and regulation of apoptosis have been documented [41, 42, 54, 55]. However, the sources of MSCs are limited. The acquisition procedures of these MSCs cause trauma to the body and are expensive, thereby limiting their use [56, 57] Compared to the other types of stem cells, human USCs are obtained from noninvasive sources and their acquisition procedures do not violate ethics, avoid immune rejection when used in autologous therapy, have a low cost of culture, and have a faster proliferative rate [32, 58]. The human USCs can differentiate into the mesoderm cell lineage, including muscle cells, adipocytes, bone cells, chondrocytes, and endothelial cells [30]. Compared to the use of MSCs in IDD therapy, USCs are a more ideal choice. When cells were treated with AKT antagonists or ERK antagonists, the antiapoptotic effect of USCs-exos was reduced. This shows that under severe ER stress, USCs-exos inhibit CHOP expression to reduce the activation of its lower-level caspase. USCs-exos inhibit excess ER stress by activating the AKT and ERK signaling pathways, suppressing UPR activation, and inhibiting CHOP expression and accumulation. These effects inhibit NPC apoptosis.

Extracellular vesicles (EVs) are membrane organelles of different sizes that are actively secreted by living cells. According to their secretion manner, they can be classified into three subgroups: apoptotic bodies, microvesicles, and exosomes [59]. Exosomes have a unique double-layer membrane structure, which makes it difficult for the substances they contain to be degraded by various enzymes in body fluids. Exosomes can be extracted and identified through their unique shape, size, and density range, as well as specific molecular markers on their surfaces. The process of IDD is associated with changes in various signal transduction mechanisms such as Wnt/*β*-catenin, MAPK, NF-*κ*B, Notch, and PI3K/Akt. These cell signaling pathways cross-influence each other to form a very complex signaling pathway network, which regulates IDD. The IDD process is accompanied by NPC apoptosis. However, the activation of the ERK signaling pathway in PI3K/Akt and MAPK inhibits cell apoptosis, promotes cell proliferation, and delays IDD. Yang et al. [60] found that estrogen can reduce the expression of caspase-3 by activating the PI3K/Akt pathway, thereby reducing the apoptosis of NPCs. Shabbir et al. [61] reported that exosomes from human bone marrow-derived MSCs can activate signal transduction pathways including Akt, Erk1/2, and STAT3 in fibroblasts. Zhang et al. [55] documented that human UCB-derived MSCs shuttle the Wnt4 protein, which induces *β*-catenin nuclear translocation and enhances epidermal cell activity. Exosomes in plasma activate the AKT and ERK pathways to promote angiogenesis and increase the expression of antiapoptotic proteins [42]. The proteins contained in exosomes have also been found to mediate the activation of AKT and ERK signals [62]. AKT and ERK signal transduction pathways are involved in the regulation of cell proliferation and migration, as well as protein synthesis, apoptosis, and metabolism [62–64]. Xu et al. found that inhibition of the AKT and ERK signaling pathways enhances ER stress and mitochondrial dysfunction-associated apoptosis [34]. We hypothesized that USCs-exos inhibit ER stress-induced apoptosis by activating the AKT and ERK signaling pathways. Elevated CHOP expression levels suppressed AKT and ERK phosphorylation. However, treatment with USCs-exos activated the AKT and ERK signaling pathways to a certain extent, thereby reducing the apoptotic rate of human NP cells. Moreover, the PI3K/AKT inhibitor (LY294002) and the ERK inhibitor (PD98059) suppressed the antiapoptotic effect of exosomes. Therefore, USCs-exos inhibit ER stress with CHOP as a downstream product.

The in vivo experiment revealed that the 21 G puncture needle can effectively cause IDD. However, the IDD degree was lower in the USCs-exos-treated group when compared to the untreated group. These findings imply that exosomes can effectively delay IDD in vivo. In vivo studies have shown that USCs-exos slow down IDD by suppressing NPC apoptosis, which is consistent with the in vitro experiment results. The expression of GRP78 and CHOP in the degenerated IVD tissue was significantly elevated, indicating that IDD is closely correlated with ER stress. In addition, the increase in pressure culture time elevated the expression levels of GRP78 and CHOP in NPCs, indicating an increase in ER stress. Equally, USCs-exos intervention suppressed the expression of GRP78, CHOP, caspase-3, and caspase-12. The injection of USCs-exos into the IVD of rats can reduce GRP78 and CHOP expression levels, indicating that exos inhibit ER stress-induced IDD. Metabolomics revealed that compared to the IVDs treated with USCs-exos, the levels of leucine (Leu), aspartic acid (Asp), and lactic acid in the purely degenerated tissue were significantly higher while the alterations in alanine (Ala) levels were small and transient. Leucine was attributed to be the amino acid residue of CHOP and aspartic acid as the amino acid residue of caspase-3, while lactic acid was the product of anaerobic glycolysis caused by ER stress. Treatment with USCs-exos caused these alterations.

The lack of nutrients in the IDD microenvironment enhances the degeneration process [65, 66]. Sufficient amounts of nutrients are required for cell survival and function. Due to the avascular nature of IVD [67–69], important nutrients (such as glucose and oxygen) can only be transferred from the capillaries of the endplate or the edge of AF to the area of the nucleus pulposus through the extracellular matrix (ECM). This translocation sharply reduces nutrient concentration in the NPCs [70–72], making it extremely difficult to maintain a healthy state of IVD. The hypoxic environment may be a normal condition of IVD cells, and the increase in oxygen concentration after IVD degeneration may be considered a pathological condition [73]. During degeneration, there is tissue angiogenesis that leads to more blood supply [74]. Studies have also reported that hypoxia promotes ECM synthesis in mammalian IVD cells [75–77]; therefore, it is essential for maintaining the normal IVD physiological structure and function. Moreover, as cells adapt, changes in oxygen concentration activate or inhibit the expression of multiple genes such as the hypoxia-inducible factor-1 (HIF-1) [78]. Hypoxia-inducible factor (HIF) is a transcription factor that responds to hypoxic tension and is one of the most important factors that directly mediate cell response to hypoxia. HIF-1 is involved in the homeostasis of NP, energy metabolism, and extracellular matrix (ECM) metabolism of NPC [79, 80]. Schipani et al. [81] reported that HIF-1 plays an important role in avascular tissue survival and increases the enzyme activity of glycolysis and metabolism under hypoxic conditions. Meng et al. [82] documented that HIF-1 knockout causes IDD. Furthermore, the interactions between HIF-1*α* and the intracellular domain of Notch protein (Notch-ICD) inhibit the differentiation of myoblasts and neural precursor cells. Hypoxia elevates the expression levels of known Notch target genes, such as Hes1 and Hey1 [83]. The Notch pathway may be involved in signal transduction during the normal functioning of IVD. IVD is a relatively complex structure in the human body. Only by using a three-dimensional (3D) structure can its in vitro growth be simulated. Rastogi et al. [84] found that the 3D structure can better maintain the IVD cell phenotype when compared to the 2D monolayer structure. Gantenbein et al. [85] also showed that 3D salt culture partially prevented the rapid loss of cell components for up to 34 days. Utilization of 3D structures in studies may change the cell's perception of physical, spatial, and biochemical factors [86]. It can also affect cell responses to inflammation and hypoxic stimulation. Studies have also shown that the 3D structure exhibits an immunomodulatory effect on the expression of catabolic genes in inflammatory and hypoxic environments [87]. The functional mechanisms of HIF-1 could be attributed to the signal transduction role of the Notch pathway. The specific mechanism has not yet been established.

Exosomes have the potential to treat IDD. MSCs-exos promote NPC proliferation and enhance the secretion of the extracellular matrix [27]. Lu et al. found that MSCs-exos inhibit NPC apoptosis by targeting the transfer of exosomal microRNA that activates the PI3K/AKT pathway [26]. Liao et al. [88] incubated MSCs-exos with NPCs induced by advanced glycation end products and confirmed that the levels of apoptosis-related markers (caspase-3 and caspase-12) were significantly suppressed. In this experiment, USCs-exos were used to intervene NPC apoptosis. The findings were consistent with the results of previous studies. Although this study revealed that USCs-exos inhibit ER stress-induced apoptosis by activating the AKT and ERK signal transduction pathways, the specific signal molecules involved have not been established. This study was limited by the parallel verification of the effectiveness of metabolomics and proteomics.

In conclusion, USCs-exos inhibited ER stress-induced apoptosis of NPCs in vivo and in vitro. Moreover, the unique advantages of USCs and their exosomes enhance their consideration as therapeutic options for IDD.

## Figures and Tables

**Table 1:** Primer sequences for quantitative real-time PCR.

Gene name	Primer sequences (5′‐3′)
CHOP	Forward (F) 5′-CTTCTCTGGCTTGGCTGACT-3′
	Reverse (R) 5′-TCTGTTTCCGTTTCCTGGTT-3′
GRP78	Forward (F) 5′-TCCTATGTCGCCTTCACTCC-3′
	Reverse (R) 5′-ATGTCTTTGTTTGCCCACCT-3′
ATF4	Forward (F) 5′-TGAAGGAGATAGGAAGCCAGA-3′
	Reverse (R) 5′-GCAGACCCACAGAGAACACC-3′
XBP1	Forward (F) 5′-ATGGATTCTGGCGGTATTGA-3′
	Reverse (R) 5′-AAAGGGAGGCTGGTAAGGAA-3′
GAPDH	Forward (F) 5′-CGACCACTTTGTCAAGCTCA-3′
	Reverse (R) 5′-AGGGGAGATTCAGTGTGGTG-3′

## Data Availability

All the data and material can be available from HongFei Xiang, WeiLiang Su, XiaoLin Wu, Zhu Guo, DongMing Xing, and BoHua Chen for reasonable request.
